# Steroids or pentoxifylline for alcoholic hepatitis (STOPAH): study protocol for a randomised controlled trial

**DOI:** 10.1186/1745-6215-14-262

**Published:** 2013-08-19

**Authors:** Ewan Forrest, Jane Mellor, Louise Stanton, Megan Bowers, Priscilla Ryder, Andrew Austin, Christopher Day, Dermot Gleeson, John O’Grady, Steven Masson, Anne McCune, David Patch, Paul Richardson, Paul Roderick, Stephen Ryder, Mark Wright, Mark Thursz

**Affiliations:** 1 Department of Gastroenterology, Glasgow Royal Infirmary, Castle Street, Glasgow G4 0SF, UK; 2 University of Southampton Clinical Trials Unit, MP 131, Southampton General Hospital, Tremona Road, Southampton SO16 6YD, UK; 3 Royal Derby Hospital, Faculty of Medical Sciences, Uttoxeter Road, Derby DE22 3NE, UK; 4 Newcastle University Medical School Framlington Place, Newcastle upon Tyne NE2 4HH, UK; 5 Liver Unit, Royal Hallamshire Hospital, Sheffield, UK; 6 Institute of Liver Studies, King’s College Hospital, Denmark Hill, London SE5 9RS, UK; 7 Liver Unit, Freeman Hospital, Freeman Road High Heaton, Newcastle upon Tyne NE7 7DN, UK; 8 Department of Hepatology, Level 2, Old Building, Bristol Royal Infirmary, Marlborough Street, Bristol BS2 8HW, UK; 9 The Royal Free, Sheila Sherlock Liver Centre, The Royal Free Hospital, Pond Street, London NW3 2QG, UK; 10 The Royal Liverpool University Hospital, Prescot Street, Liverpool L7 8XP, UK; 11 Public Health Sciences and Medical Statistics, University of Southampton, C floor South Academic Block, Southampton General Hospital, Southampton SO16 6YD, UK; 12 Department of Gastroenterology, Queen’s Medical Centre campus, Nottingham University Hospitals NHS Trust, Nottingham NG7 2UH, UK; 13 Southampton General Hospital, Tremona Road, Southampton SO16 6YD, UK; 14 Hepatology Section, Imperial College, Norfolk Place, London, Paddington W2 1NY, UK

**Keywords:** Factorial design, Alcoholic hepatitis, Prednisolone, Pentoxifylline, Maddrey’s discriminant function (DF), Glasgow alcoholic hepatitis score (GAHS)

## Abstract

**Background:**

Alcoholic hepatitis is the most florid presentation of alcohol-related liver disease. In its severe form, defined by a Maddrey’s discriminant function (DF) ≥32, the 28-day mortality rate is approximately 35%. A number of potential treatments have been subjected to clinical trials, of which two, corticosteroids and pentoxifylline, may have therapeutic benefit. The role of corticosteroids is controversial as trial results have been inconsistent, whereas the role of pentoxifylline requires confirmation as only one previous placebo-controlled trial has been published.

**Methods/design:**

STOPAH is a multicentre, double-blind, factorial (2 × 2) trial in which patients are randomised to one of four groups:

1. Group A: placebo / placebo

2. Group B: placebo / prednisolone

3. Group C: pentoxifylline / placebo

4. Group D: pentoxifylline / prednisolone

The trial aims to randomise 1,200 patients with severe alcoholic hepatitis, in order to provide sufficient power to determine whether either of the two interventions is effective. The primary endpoint of the study is mortality at 28 days, with secondary endpoints being mortality at 90 days and 1 year.

**Discussion:**

STOPAH aims to be a definitive study to resolve controversy around the existing treatments for alcoholic hepatitis. Eligibility criteria are based on clinical parameters rather than liver biopsy, which are aligned with standard clinical practice in most hospitals. The use of a factorial design will allow two treatments to be evaluated in parallel, with efficient use of patient numbers to achieve high statistical power.

**Trial registration:**

EudraCT reference number: 2009-013897-42

ISRCTN reference number: ISRCTN88782125

## Background

Alcohol-related illness places an enormous burden upon health services. A recent review of the burden of liver disease in Europe confirms the prominence of alcohol as the most important cause of cirrhosis and liver-related mortality in Europe. In the UK it has been estimated by the Royal College of Physicians of London that the inpatient costs in 1998-1999 arising from the consequences of alcohol misuse were as high as £2.9 billion [1]. Overall, alcohol-related deaths in the UK have more than doubled since 1979, and in Scotland have increased by 236% between 1980 and 2002 [2,3]. Throughout the UK deaths from cirrhosis have risen dramatically between 1987 and 1991 [4]. Alcohol-related liver disease (ALD) accounts for majority of alcohol-related deaths in the UK [5]. While many patients presenting with alcoholic liver disease will have cirrhosis, as many as 60% will have evidence of an alcohol-related hepatitis [6]. Alcoholic hepatitis is the most florid manifestation of alcohol-related liver disease, but is potentially reversible. However the short-term mortality of alcoholic hepatitis is particularly high among those with indicators of severe disease. The 28-day mortality of patients who have a Maddrey’s discriminant function (DF) ≥32 is 30% to 40% [7-9]. The 28-day mortality of patients who have a Glasgow Alcoholic Hepatitis Score (GAHS) ≥9 is approximately 60% [10]. Alcoholic hepatitis affects a relatively young population (average age, 50 years; patients may present in their 20s and 30s). Despite the increasing prevalence and the severity of this disease, there is no consistency in its management.

Since 1971 there have been 13 randomised studies and four meta-analyses investigating the role of corticosteroid therapy for alcoholic hepatitis [11]. Despite this apparent wealth of evidence, controversy persists. There remains deep division with regard to the use of corticosteroids. Advocates of the treatment cite significant improvement in the short to medium term mortality, while detractors cite the risks of sepsis and gastrointestinal haemorrhage with corticosteroid therapy. Many of the published studies have been plagued by widely varying inclusion and exclusion criteria. The largest placebo-controlled study treated 90 patients and found no benefit with prednisolone compared with a similar placebo-treated group [12]. This study was hampered by the inclusion of patients with both moderate and severe alcoholic hepatitis, as well as end-stage alcoholic liver disease. In the only study to require histological confirmation of alcoholic hepatitis in all patients, prednisolone was associated with a short-term improvement in mortality in patients, although this benefit was not apparent after 2 years [13,14]. However, on review of the published studies, none of these reach an adequate statistical power to make a statement with 80% confidence. The most recent meta-analysis by the Cochrane Group, of all of the available trials, demonstrated that although there was a trend of benefit with corticosteroids, the results were not statistically significant (*P*=0.2) [15]. However, a re-analysis of the five most recent studies indicated a significant benefit from corticosteroid [16]. In this study, patients with DF ≥32 treated with prednisolone had a 28-day mortality of 20%, as opposed to a mortality of 34.3% among placebo-treated patients (*P*=0.006).

Pentoxifylline has also recently been studied in the treatment of alcoholic hepatitis. It is believed to act, in part, by inhibiting the synthesis of the pro-inflammatory cytokine tumour necrosis factor alpha. There has been one randomised placebo controlled trial which showed significant benefit [17]. One hundred patients were enrolled, all with a DF >32. Pentoxifylline was administered for 4 weeks, at a dose of 400 mg TDS. A total of 12/49 (24.5%) in the pentoxifylline group died compared to 24/52 (46.1%) in the placebo group during the index hospitalisation (*P*=0.037). The principal benefit for the agent appeared to be a reduction of deaths attributed to hepatorenal syndrome.

One study has compared prednisolone with pentoxifylline and suggested a survival benefit with pentoxifylline at 3 months (85.3% compared to 64.7%; *P*=0.04). Hepatorenal failure again appeared to be less in the pentoxifylline group. However, this was a small study with only 34 patients in each group [18]. The comparison of prednisolone alone against prednisolone plus pentoxifylline has been assessed in two studies. The COPE Trial studied 70 patients and found similar 28-day survival for steroid treated patients with or without pentoxifylline (73.5% and 72.2%, respectively) [19]. A further study published in abstract form was similarly unable to show a difference in the rate of 28-day mortality [20]. In addition, one study looked at the effect of switching from prednisolone to pentoxifylline in those patients who showed no evidence of a response after 7 days of steroid treatment [21]. Again there was no difference in mortality between these groups.

The primary objective of this study is to determine whether corticosteroids or pentoxifylline reduce the mortality associated with severe alcoholic hepatitis at 28 days, 90 days and 1 year. In order to avoid the controversies caused by underpowered studies in this field, we aim to conduct a well-powered definitive study.

## Methods/design

The study design is a multicentre, double-blind, factorial (2 × 2) trial in which patients are randomised to one of four groups:

1 Group A: placebo / placebo

2 Group B: placebo / prednisolone

3 Group C: pentoxifylline / placebo

4 Group D: pentoxifylline / prednisolone

Patients admitted to participating hospitals with a clinical diagnosis of alcoholic hepatitis are screened using the inclusion and exclusion criteria below. Clinical and demographic data are transmitted to the TENALEA web-based registration/randomisation service, which randomises the patient to a treatment group stratified by disease severity and geographical region.

### Clinical study endpoints

The primary endpoint is mortality at 28 days; this time point represents the end of the peak period of mortality for alcoholic hepatitis and is consistent with other trials in the field [22]. Secondary endpoints will look at mortality at 90 days and 1 year, outcome relative to GAHS, rates of recidivism, hospital re-admission rates for liver or non-liver related events, rates of gastrointestinal haemorrhage and sepsis, and rates of new or recurrent renal failure (serum creatinine >500 μmol/L or requiring renal support) and any other adverse events or serious adverse events recorded using NCI CTCAE v4.0). In addition incremental NHS costs and quality of life (QOL) using SF36 and EQ-5D will be collected to facilitate health economic analysis.

### Patient population

Patients, at least 18 years of age, with a clinical diagnosis of alcoholic hepatitis on admission to hospital are considered for inclusion in the study. Eligibility criteria (Appendices A & B) are used to select those patients with severe alcoholic hepatitis (defined by a Maddrey’s DF >32) and to exclude patients with other hepatological diagnoses. As the trial is being conducted in more than 60 hospitals across the UK, many of which do not have access to transjugular liver biopsy, it was decided not to make liver histology an entry criteria. However, in order to exclude patients with decompensated cirrhosis, a strict time limit was set on the duration of jaundice.

In the majority of published trials, patients with gastrointestinal bleeding, sepsis and renal failure prior to randomisation have been excluded from the study. In this trial, patients with gastrointestinal bleeding are eligible for randomisation once they have been stabilised for 48 h. All patients are carefully screened for infection, by clinical examination, chest radiology, blood and urine cultures and, if found to have evidence of sepsis, are treated with appropriate antibiotics for a minimum of 2 days. Once the local investigator considers the infection to be under control, the patient is then eligible for randomisation. This strategy is justified by the recent observation that the outlook of patients with AH receiving corticosteroids is unaffected by their having had a recent infection treated with antibiotics [23]. Patients who are oligo-anuric (urine output < 400 mL / 24 h), have a creatinine >500 uM/L or who require renal support, are given appropriate resuscitation therapy for up to 1 week. These patients may then be re-screened and considered for randomisation, once they meet eligibility criteria.

### Consent

Patient information sheets are given to potential patients at least 24 h before consent is sought. Potential patients for the trial who present with hepatic encephalopathy may be unable to consent for themselves, but are not excluded from the trial. Special arrangements are in place to ensure that the interests of such patients are protected. When considering a patient who is unable to consent for themselves for suitability for the trial, the decision on whether to consent to, or refuse, participation in a trial is taken by a ‘legal representative’ who is independent of the research team and who acts on the basis of the person’s presumed wishes. The legal representative is generally the next of kin but where the next of kin is not available or unwilling to take the decision, a hospital-appointed medical practitioner unconnected to the trial is asked to provide consent. The consent given by the legal representative remains valid in law until such time as the patient recovers capacity. At this point, the patient is informed about the trial and asked to decide whether or not they want to continue in the trial, and consent to continue is sought from the patient themself.

### Randomisation

After consent is given, patients are registered in the trial via TENALEA, a web-based registration and randomisation system, and then undergo screening assessments. If eligible for the study, patients are randomised via the ALEA system to a study treatment group, which is blinded to the site staff and the patient, by means of a unique four-digit patient pack number.

Randomisation is block stratified and performed using the following two stratification factors:

1. Geographic region (12 in total)

2. Risk group: either high or intermediate risk (high risk is defined as either sepsis or history of GI bleeding in the previous 7 days or creatinine >150 μmol/L or any combination of the these; intermediate risk is defined as no sepsis and no history of GI bleeding in the previous 7 days and creatinine ≤150 μmol/L).

### Intervention

Each patient is provided with two bottles labeled ‘Bottle A’ and ‘Bottle B’ containing the investigational medical products. Bottle A contains opaque gelatin capsules filled either with tablets of pentoxifylline 400 mg or identical placebo capsules filled with microcrystalline cellulose. Patients are instructed to take one capsule from bottle A three times daily. Bottle B contains opaque gelatin capsules filled either with tablets of prednisolone 40 mg or identical placebo capsules filled with microcrystalline cellulose. Patients are instructed to take one capsule from bottle B once daily. Both medications are administered for 28 days.

Alcohol withdrawal therapy is administered if required. All patients receive supportive nutritional therapy with nutritional supplements in the first instance. If they are unable to take these, they are offered enteral nutrition via a nasogastric tube with the aim of providing 35 to 40 kcal/kg/day non-protein energy with 1.5 g/kg/day protein.

### Evaluations during and after treatment

Patients are evaluated while an inpatient on treatment days 7, 14, 21, and 28 and at each time point recordings made of vital signs, WHO performance status, concomitant medication and adverse events. Blood samples are taken for liver function tests, prothrombin time, full blood count, urea and creatinine. Patients are assessed for the presence of hepatic encephalopathy and the occurrence of GI bleed or sepsis in previous 7 days. If patients are discharged from hospital before the end of treatment, assessments are made at 28 days by telephone interview. A complete schedule of procedures is given in Additional file 1 (Trial Procedures; Schedule of procedures).

After discharge from hospital, patients are evaluated at 90 days and at one year. At each time point recordings are made of vital signs, WHO performance status, concomitant medication and adverse events. Blood samples are taken for liver function tests, prothrombin time, full blood count, urea and creatinine. Patients are assessed for the presence of hepatic encephalopathy and the occurrence of GI bleed or sepsis since previous assessment. In addition, patients are asked to complete a quality of life questionnaire (Short Form 36 and EQ-5D) and an assessment is made of their current alcohol consumption. At registration patients are asked to consent to follow-up via medical research information service, now called the NHS Information Centre Data Linkage service. This ensures that if patients are lost to follow-up should the patient die, we would obtain their date of death for analysis via this service.

### Statistical considerations

#### Power calculation

In order to estimate the required trial sample size a power calculation was performed in nQuery Advisor using a two group continuity corrected χ^2^ test and the following parameters:

• Power = 90% (to allow for secondary outcomes)

• Two-sided significance level of 5%

• Estimated 28-day mortality rate in each treatment group is given in Table 1.

**Table 1:** Estimated 28 day mortality rates for power calculation

		**Pentoxifylline**		
		**Yes**	**No**	**Total**
Prednisolone	Yes	17%^a^ Group D	25% Group B	21%
	No	25% Group C	35% Group A	30%
	Total	21%	30%	

^a^Estimated assuming no interaction, that is multiplicative independent effects.

Based on a reduction in the 28-day mortality rate at the margins from 30% to 21%, a sample size of 513 per group of single agent *versus* no single agent would be required. Thus in total the trial would require 1,026 patients. We have allowed for an approximate 10% withdrawal/lost to follow-up rate and will therefore aim to recruit 1,200 patients to the study, with patients being evenly allocated to each treatment arm.

The sample size for this trial has not been powered to assess for any observed treatment interaction and in fact assumes no interaction between the two treatments (that is, that receiving prednisolone in addition to pentoxifylline does not change the effect of pentoxyfylline and *vice versa*). To be able to assess the interaction of two treatments, while keeping power at 90%, generally requires an increase in the sample size by four-fold [24]. As assessing the size of any interaction was not of primary interest as it was assumed to be small or non-existent, it was deemed appropriate to not power for assessing an interaction.

### Statistical analyses

Analysis will be on the basis of intention to treat (ITT). In order to determine the efficacy of prednisolone, the 28-day mortality rate in those treated with prednisolone (that is, Groups B and D) will be compared with the mortality rate in control groups (Groups A and C). Similarly, the efficacy of pentoxifylline will be assessed by comparing the 28-day mortality rate in Groups C and D with the mortality rate in Groups A and B.

Although the study is not powered to detect a difference between two active treatments and single agent therapy, we will make this comparison for 28-day mortality rates. Logistic regression will be used to compare 28-day mortality between the treatment groups. The impact of pre-treatment variables such as gastrointestinal bleeding, sepsis or renal impairment on admission will be estimated by adding these covariates to the logistic regression analysis. Mortality rates at 90 days and 12 months will be compared using the same strategy. All analyses will be adjusted for the stratification variables and assessed at the two-sided 5% significance level. However, the impact of recidivism and hospital re-admission will also be assessed in relation to these outcomes.

Overall mortality will also be analysed as a secondary endpoint using the method of Kaplan-Meier and a cox-proportional hazards model adjusting for the same variables as explained above.

Rates of gastrointestinal bleeding, sepsis and renal failure will be summarised over time and by arm.

Stata for Windows (StataCorp) and SAS (SAS insititute inc) will be the statistical packages of choice. The study will be reported in accordance with the CONSORT (Consolidated Standards of Reporting Trials) statement.

### Trial organisation

#### Trial funding

The trial is funded by the UK National Institute for Health Research Health Technology Assessment Board (NIHR-HTA): 08/.14/44 http://www.hta.ac.uk/project/2015.asp.

### Trial management group

The Trial Management Group (TMG) is composed of a chairperson, vice chair, trial statistician, trial manager and 12 of the principal investigators. The TMG is responsible for approval of the trial design, reviewing and advising on trial recruitment, reviewing the final results, approving publications and approval of secondary studies.

### TMG executive group

The TMG Executive Group (TMG EG) is composed of the chairperson and vice chair of the TMG, one additional principal investigator, the trial statistician and the trial manager. The TMG EG is responsible for the conduct of the study, implementing decisions of the full TMG and ensuring that recruitment and data collection occur at an acceptable rate.

### Data monitoring and ethics committee

The Data Monitoring and Ethics Committee (DMEC) is composed of a clinical chair, an independent hepatologist and an independent statistician. The DMEC, in accordance with ICH-GCP guidelines is responsible for safeguarding the interests of trial participants, monitoring the main outcome measures including safety and efficacy, and monitoring the overall conduct of the trial. The DMEC receive, on a periodical basis, unblinded data on trial recruitment and data quality, outcome measures and safety data. The DMEC is required to assess emerging external evidence which might influence the ethical position of the trial. The committee is also expected to advise on any protocol modifications and whether the trial (or specific treatment groups) should be stopped early.

In agreement with the DMEC pre-planned interim analyses will be carried out after 200, 400 and 800 patients have reached the primary endpoint. Pre-specified stopping guidelines based on the Peto-Haybittle rule will be used at these interim looks. Namely, a two-sided *P* value <0.001 for harm or benefit (symmetrical stopping boundary) would indicate the DMEC recommend that the study or certain trial arms should be stopped. This method is being used because the treatments are already in use in practice and should evidence of harm or benefit arise, it needs to be convincing to ensure that others will change their practice accordingly. A *P* value of 0.001 is sufficiently small that the sample size does not need to be adjusted for these interim analyses [25].

### Ethical approval

Ethical approval was provided by the Wales Research Ethics Committee (Reference 09/MRE09/59) and a clinical trials authorisation issued by the Medicines and Healthcare products Regulatory Agency (MHRA) (Reference 11709/0227/001-0001).

## Discussion

An objective review of the literature on the treatment of alcoholic hepatitis inevitably encounters the controversy over the use of corticosteroids and the lack of confirmatory data on the use of pentoxifylline. Current international guidelines recommend the use of corticosteroids except in patients with sepsis, where pentoxifylline is the first choice of treatment [26]. These recommendations are attributed a lower grade of evidence acknowledging the controversies outlined above. Given the importance of this condition, a definitive large trial was required to settle the dilemma. If corticosteroids prove to be successful in this trial, it would still be reasonable to expect a mortality rate of 20% in treated patients based on the analysis of recent trials. There will remain an unmet medical need in this field, but at least future trials will legitimately use a steroid (or pentoxifylline) control arm based on solid evidence.

At the heart of the controversy over the use of corticosteroids is susceptibility to infection. Although 40 mg daily of prednisolone is considered a moderate dose, this group of patients is already highly susceptible to infection and corticosteroids are likely to exacerbate this problem. The emergence of infection during treatment is associated with a significant increment in the mortality rate [23].

Optimally, all patients enrolled in a treatment trial for alcoholic hepatitis would have a liver biopsy to confirm the diagnosis histologically [26]. However, the group of patients addressed by this study invariably have a coagulopathy and cannot be subjected to percutaneous biopsy. Transjugular liver biopsy is only available in specialist centres but alcoholic hepatitis is not usually considered as an indication for transfer to tertiary care. In addition even in specialist centres adequate histological samples may not be obtained in up to 4% of patients and biopsies may not be undertaken for a week after admission [27]. Previous studies which have a defined minimum level of bilirubin have confirmed histological features of alcoholic hepatitis in 98% of patients [28] and 96% of patients with acute-on-chronic liver alcoholic liver disease [29]. It was therefore decided to use strict clinical criteria including a minimum threshold of bilirubin to define the eligible patient group. This is a pragmatic decision which aligns the trial with the clinical practice that it should influence. Histology data will be collected from participating centres which have access to transjugular liver biopsy and used to validate the clinical criteria.

The primary endpoint of ‘mortality at 28 days’ was selected to align the study with previous trials in this area. As one previous trial showed important divergence between treatment groups after the 28-day time point arising due to increased rates of infection in the steroid arm, we believe that the 90-day mortality comparison will also be of interest to clinicians [30]. Previous studies have demonstrated that mortality beyond this time point is predominantly influenced by recidivism rather than the initial treatment choice [20].

Factorial 2 × 2 design is a well-established technique for concurrently assessing the benefit of two active treatments and is particularly appropriate in this situation where doubt exists over the efficacy of both medications. Factorial design trials are generally more powerful in detecting treatment effects unless there is a strong synergistic or inhibitory interaction between the two active medications. This is not considered to be likely in the current trial and it has rarely been observed in published trial results [31]. The two studies which have compared treatment with corticosteroids alone against the combination of corticosteroids and pentoxifylline have failed to demonstrate any difference in mortality rate between the two groups despite reasonable patient numbers. If the same is found in the current study then the assumption around no interaction between prednisolone and pentoxifylline may be challenged, in which case the trial will need to be analysed as a four-arm, which will inevitably reduce the study power and increase the width of any estimated confidence intervals.

## Trial status

The trial is currently recruiting. Recruitment commenced on 1 February 2011 and will finish on 28 February 2014.

## Appendix A: Inclusion criteria

• Aged 18 years or older

• Clinical alcoholic hepatitis:

 - Serum bilirubin >80 μmol/L

- History of excess alcohol (>80 g/day male, >60 g/day female) to within 2 months of randomisation

• Less than 4 weeks since admission to hospital

• Discriminant function (DF)* ≥32

• Informed consent

• * DF = 4.6 × prothrombin time + (serum bilirubin (μmol/L) / 17.1) Prothrombin time (PT) = PT_PATIENT_ - PT_CONTROL_PT_CONTROL_ is defined as the midpoint value at each site; this mean value may be updated on a weekly or monthly basis.

## Appendix B: Exclusion criteria

• Abstinence of >2 months prior to randomisation

• Duration of clinically apparent jaundice >3 months

• Other causes of liver disease including:

◦ Evidence of chronic viral hepatitis (Hepatitis B or C)

◦ Biliary obstruction

◦ Hepatocellular carcinoma

• Evidence of current malignancy (except non-melanotic skin cancer)

• Previous entry into the study, or use of either prednisolone or PTX within 6 weeks of admission

• AST >500 U/L or ALT >300 U/L (not compatible with alcoholic hepatitis)

• Patients with a serum creatinine >500 μmol/L or requiring renal support

• Patients dependent upon inotropic support (adrenaline or noradrenaline). Terlipressin is allowed

• Active gastrointestinal bleeding

• Untreated sepsis

• Patients with known hypersensitivity to pentoxifylline, other methyl xanthines, or any of the excipients

• Patients with cerebral haemorrhage, extensive retinal haemorrhage, acute myocardial infarction (within the last 6 weeks) or severe cardiac arrhythmias (not including atrial fibrillation)

• Pregnant or lactating women

## Competing interests

The authors declare that they have no competing interests.

## Authors’ contributions

EF, AA, CD, DG, JO’G, AM, DP, SR, MW and MT participated in the conception and design of the study. JM, LS, MB, SM, PRi and PRo designed the study. EF, JM, SR, LS and MT wrote the manuscript. All authors read and approved the manuscript.

## Supplementary Material

Additional file 1: Table S1 Schedule of assessments. Click here for file
